# General Practitioners and Breast Surgeons in France, Germany, Netherlands and the UK show variable breast cancer risk communication profiles

**DOI:** 10.1186/s12885-015-1281-2

**Published:** 2015-04-09

**Authors:** Claire Julian-Reynier, Anne-Deborah Bouhnik, D Gareth Evans, Hilary Harris, Christi J van Asperen, Aad Tibben, Joerg Schmidtke, Irmgard Nippert

**Affiliations:** 1 Institut Paoli-Calmettes, UMR_S 912, 232 Boulevard Sainte Marguerite, 13009 Marseille, France; 2 INSERM, UMR_S 912, Marseille, France; 3 Aix-Marseille Université, UMR_S 912, Marseille, France; 4 Department of Genomic Medicine, The University of Manchester, Manchester Academic Health Science Centre, St. Mary’s Hospital, Oxford Road, Manchester, M13 9WL UK; 5 GenEd Coordinating Centre, University of Manchester, Manchester, UK; 6 Department of Clinical Genetics, Leiden University Medical Centre, Leiden, The Netherlands; 7 Institute of Human Genetics, Hannover Medical School, Hannover, Germany; 8 Women’s Health Research, Münster Medical School, Münster, Germany

**Keywords:** Risk communication, Breast cancer, Physicians’ attitudes, International study, General Practitioners, Breast Surgeons

## Abstract

**Background:**

No information is available on the attitudes of General Practitioners (GPs) and Breast Surgeons (BSs) to their delivery of genetic, environmental and lifestyle risk factor information about breast cancer. The aim of this study was to describe the Breast Cancer Risk Communication Behaviours (RCBs) reported by GPs and BSs in four European countries and to determine the relationships between their RCBs and their socio-occupational characteristics.

**Methods:**

Self-administered questionnaires assessing breast cancer risk communication behaviours using vignettes were mailed to a sample of Breast Surgeons (BS) and General Practitioners (GP) working in France, Germany, the Netherlands, and the UK (N = 7292). Their responses to questions about the risk factors were first ordered and compared by specialty and country after making multivariate adjustments. Rather than defining a standard Risk Presentation Format (RPF) *a priori,* the various RPFs used by the respondents were analyzed using cluster analysis.

**Results:**

Family history and hormonal replacement therapy were the risk factors most frequently mentioned by the 2094 respondents included in this study. Lifestyle BC risk factors such as obesity and alcohol were rarely/occasionally mentioned, but this point differed (p < 0.001) depending on the country and the specialty of the providers involved. Five distinct RPF profiles including the numerical/verbal presentation of absolute/relative risks were identified. The most frequently encountered RPF (34.2%) was characterized by the fact that it included no negative framing of the risks, i.e., the probability of not developing cancer was not mentioned. Age, specialty and country of practice were all found to be significant determinants of the RPF clusters.

**Conclusions:**

The increasing trend for GPs and BSs to discuss lifestyle risk factors with their patients suggests that this may be a relevant means of improving breast cancer prevention. Physicians’ risk communication skills should be improved during their initial and vocational training.

## Background

Multiple risk factors contribute to the occurrence of breast cancer. It has been clearly established by now that lifetime exposure to genetic, environmental and lifestyle risk factors play an important role in the aetiology of this disease. Convincing or at least strong evidence has been presented showing that genetic mutations such as *BRCA1/BRCA2* or a family history of breast cancer, hormonal factors increasing the duration of exposure to oestrogens such as early menarche/late menopause/hormonal replacement therapy/oral contraceptives, exposure to ionising radiation at an early age (especially <30 years) and a high breast density can all contribute to breast cancer [1-3]. Decreasing the duration of exposure to oestrogens by practising breast-feeding for at least 25 months has been found to decrease the rates of occurrence of breast cancer [4]. Lifestyle risk factors such as a high alcohol consumption and a high energy intake (obesity after menopause or weight gain after 18 years of age), have been found to contribute significantly to the occurrence of breast cancer, whereas smoking is a risk factor on which more evidence is required [1-3]. Interactions between these factors have also been described, especially in *BRCA1/2* mutation carriers [5].

As soon as the first genes predisposing their carriers to hereditary breast/ovarian/colorectal cancer were identified, cancer genetic clinics were organised in many industrialized countries to inform people at risk and their families [6-11]. Since an increasingly large target population is becoming eligible for cancer genetic counselling/genetic testing, many primary care providers and General Practitioners are now also being expected to deliver relevant information to individuals at risk [12,13]. One of the key issues which needs to be addressed by healthcare providers is how to communicate information about patients’ genetic/family risks and other significant changeable risk factors for preventive purposes.

As far as we know, no information is available so far about GPs’ and breast specialists’ attitudes to delivering genetic, environmental and lifestyle risk factor information about breast cancer. The state of the art and the effectiveness of risk communication have been reviewed extensively [14], especially in the context of genetic cancer risks [15-17]. The “best” theoretical risk communication standards have been reported to consist in presenting numerical information with or without a verbal interpretation, presenting both the absolute and relative risks, and discussing the positively and negatively framed event [14]. However, the authors of empirical studies on the pragmatic and contextual aspects of risk communication have pointed out that even personally tailored breast cancer risk information may not account completely for the specific factors that patients hold to be the most relevant [18] and that the complexity of the task of providing patients with medical information obliges practitioners to make choices [19].

The aim of this study was first to ascertain how General Practitioners (GPs) and Breast Surgeons (BSs) practicing in several European countries regarded their Breast Cancer Risk Communication Behaviours (RCBs), and to determine the relationship between their RCBs and their socio-occupational characteristics.

## Methods

### The InCRisC study

The International Cancer Risk Communication Study (InCRisC study) was a multicentre European research project designed to describe risk communication practices and the management of familial breast cancer in primary care. It was carried out in 2010 in four European countries (France, Germany, the Netherlands and the UK). Data collection was organised separately in each country by the co-authors involved in the project (CJR for France, JS and IN for Germany, CJA and AT for the Netherlands, and DGE and HH for UK). Questionnaires taking about 25 min to complete were posted to a sample of 3999 GPs and a sample of 3293 breast surgeons in each of these four countries. The international study methodology was reviewed and approved by the German Federal Ministry of Education and Research «Ethical, Legal and Social Implication of Biomedical Research Programme» which funded the project. Each country was in line with its own national regulation. The review procedure included the assessment of ethical issues, informed consent, confidentiality and regulation to data access. A detailed description of the study design has been published elsewhere [20]. Practitioners who were not consulted by any breast cancer patients during the year prior to the survey were excluded from the analysis.

### Questionnaire

**Figure 1:**
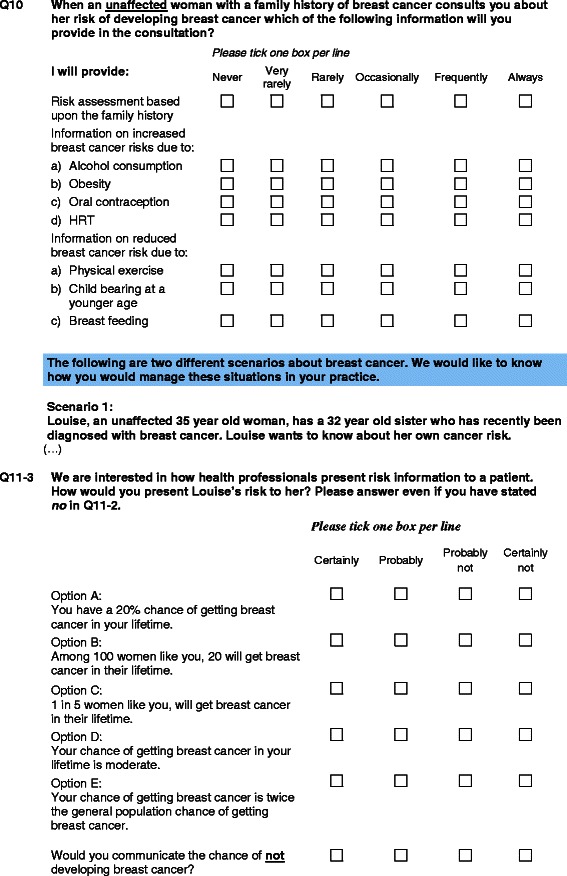
**Questions adressed to doctors on risk communication –InCRisC study.**

The questionnaire included 70 questions based on a set of clinical vignettes about women with a family history of breast cancer and the initial steps in the *BRCA1/2* genetic testing process. Here we present only the variables addressed in the questionnaire on the topic of risk communication (Figure 1).

### Communication about breast cancer risk factors

Practitioners were asked what risk information they provided during their consultations when unaffected women with a family history of breast cancer consulted them about their risk of developing breast cancer: risk assessment based on the family history, increased risks due to alcohol consumption, obesity, oral contraception, and/or HRT, and risk prevention based on physical exercise, child bearing at a younger age, and/or breast feeding.

Answers were given on a 6-item Likert scale (never; very rarely; rarely; occasionally; frequently; always). Scores ranging from 0 (never) to 5 (always) were computed on each of these 8 variables. The questions used are given in Figure 1-Q10.

### Risk presentation formats

In the first scenario presented to the physicians (Figure 1-Q11), they were asked how they would present the breast cancer risks involved to their patients. Six different formats were proposed: in the first four formats, absolute risks were presented, while the last two corresponded to relative risks and negative framing, respectively. Answers to these 6 formats were given on a 4-item Likert scale (certainly; probably; probably not; certainly not); binary outcomes were analysed, in which the responses certainly/probably were compared with the other possible answers. The first three types of presentation were then grouped together, as they all corresponded to the numerical presentation of the absolute risks.

### Socio-demographics and occupational characteristics

These characteristics included all the participants’ gender, age, and number of years of practice. GPs were also asked approximately how many patients consulted them per week, while specialists were asked about their speciality and the number of newly diagnosed breast cancer patients who had consulted them during the previous year.

### Statistical analysis

Chi^2^ and Fisher’s exact tests were used in the univariate comparisons on categorical data and Student’s t-tests on continuous data. Multivariate adjustment was performed using analyses of variance (ANOVA) on quantitative dependent variables. Logistic regressions were performed in order to compare the physicians’ profiles identified in the cluster analysis in terms of their risk presentation formats. In the multivariate analyses, systematic adjustments were made for age, gender, country and specialty.

Rather than defining the formats used by physicians *a priori*, we decided to identify their profiles statistically, based on their answers to the questions about their risk presentation practices, using cluster analysis.

The advantage of this method was that the profiles could be tested without making any *a priori* hypotheses about them. The cluster analyses were performed using the conventional agglomerative hierarchical procedure [21] to attribute individuals to clusters so that those belonging to the same cluster were as similar as possible and each cluster was clearly distinct from the others. In this way, a set of contrasting profiles reflecting the physicians’ various risk presentation practices was set up. In order to avoid fixing the number of clusters *a priori*, an automatic procedure was used to select the best distributions (from 3–10) using Ward’s method [22].

An overall score on the communication of risk factors other than family history and HRT was calculated by summing together all the risk factor scores except for family history. This score ranged from 0 to 30.

The Statistical Package for Social Science (SPSS/PC released 18.0) was used to perform all the statistical analyses (SPSS Inc., Chicago, IL, USA).

## Results

### Study sample

The final sample analysed (N = 2094) consisted of 1189 GPs and 905 breast surgeons.

The response rates differed significantly from one country to another (p < 0.001): 40.6% in France, 28.4% in Germany, 38.6% in the Netherlands, and 30.1% in the United Kingdom. Details of the response rates obtained in these countries have been presented elsewhere [20].

Among the respondents, 7 GPs and 22 Breast Surgeons were excluded from the analysis because they declared that they had not been consulted by any breast cancer patients during the previous year.

### Description of the sample

Doctors’ socio-occupational characteristics differed among the four countries (Table 1).

**Table 1:** **Characteristics of the sample – InCRisC (N = 2094)**

	France	Germany	The Netherlands	UK	Total
***GPs***	*n = 269*	*n = 449*	*n = 264*	*n = 207*	*n = 1189*
	Mean (SD)	Mean (SD)	Mean (SD)	Mean (SD)	Mean (SD)
Age*	48.5 (9.2)	54.2 (7.7)	49.5 (8.0)	45.1 (9.2)	50.3 (9.0)
Number of years of medical practice*	21.9 (9.1)	25.0 (8.3)	20.1 (8.7)	16.5 (9.5)	21.7 (9.3)
	n (%)	n (%)	n (%)	n (%)	n (%)
Gender male*	170 (63.2)	295 (65.7)	175 (66.3)	85 (41.1)	725 (61.0)
Weekly number of consultations > 100*	144 (53.5)	411 (91.5)	171 (64.8)	131 (63.3)	857 (72.1)
***Breast surgeons***	*n = 168*	*n = 458*	*n = 122*	*n = 157*	*n = 905*
	Mean (SD)	Mean (SD)	Mean (SD)	Mean (SD)	Mean (SD)
Age*	52.1 (10.1)	50.7 (7.4)	46.7 (8.9)	48.3 (8.7)	50.0 (8.6)
Number of years of medical practice*	25.8 (10.1)	22.3 (7.9)	16.3 (7.9)	14.8 (8.9)	20.8 (9.5)
	n (%)	n (%)	n (%)	n (%)	n (%)
Gender male*	147 (87.5)	250 (54.6)	85 (69.7)	105 (66.9)	587 (64.9)
BCs newly diagnosed during the last year > 100*	40 (16.6)	105 (22.9)	21 (17.2)	75 (47.8)	241 (26.6)

BC: Breast Cancer.

*p < 0.001.

### Informing patients about breast cancer risks

BS informed their patients more frequently than GPs about all the risk factors except for oral contraception and HRT, on which GPs obtained higher scores (Table 2). Information about all the risk factors studied here was communicated differently between countries, but the differences were much stronger in the case of risk factors other than family history and HRT (Table 3). German practitioners obtained the highest scores, whereas Dutch practitioners had the lowest scores on risk factors other than family history and HRT; French and British practitioners obtained intermediate scores. HRT yielded higher scores than the other risk factors in all the countries involved, followed by family risks. Oral contraception ranked third in all the countries except for Germany.

**Table 2:** **Risk factors presented to patients, depending on practitioners’ (GPs’ and Breast Surgeons’) speciality InCRisC (N=2094)**

	GP (n=1189)		BS (n=905)		
	Mean score ± SD	Rank	Mean score ± SD	Rank	p-value*
Family history of BC	3.4 ± 1.5	2	4.1 ± 1.2	1	<.001
HRT	3.9 ± 1.1	1	3.8 ± 1.3	2	0.029
Oral contraception	3.2 ± 1.5	3	2.6 ± 1.7	8	<.001
Breast feeding	2.8 ± 1.6	4	3.1 ± 1.5	5	<.001
Physical exercise	2.6 ± 1.7	5	3.3 ± 1.7	3	<.001
Obesity	2.5 ± 1.6	6	3.2 ± 1.6	4	<.001
Childbearing at a younger age	2.2 ± 1.6	8	2.7 ± 1.6	7	<.001
Alcohol	2.3 ± 1.7	7	2.8 ± 1.7	6	<.001
Risk factors for BC other than family history and HRT (sum)	15.5 ± 7.8		17.7 ± 7.5		<.001

BC: Breast Cancer/BS: Breast Surgeon/HRT: Hormonal Replacement Therapy.

*Adjusted for age, gender and country using multifactor ANOVAs.

**Table 3:** **Risk factors presented by the practitioners (GPs and Breast Surgeons), depending on the country of origin – InCRisC (N=2094)**

	France (n=437)		Germany (n=907)		NTL (n=386)		UK (n=364)		
	Mean ± SD	Rank	Mean ± SD	Rank	Mean ± SD	Rank	Mean ± SD	Rank	p-value*
Family history of BC	3.8 ± 1.3	1	3.7 ± 1.5	2	3.5 ± 1.4	2	3.7 ± 1.4	2	0.023
HRT	3.8 ± 1.3	1	3.9 ± 1.2	1	3.7 ± 1.2	1	3.9 ± 1.0	1	0.002
Oral contraception	2.8 ± 1.7	3	2.8 ± 1.7	7	2.9 ± 1.5	3	3.3 ± 1.4	3	<.001
Breast feeding	2.8 ± 1.7	3	3.3 ± 1.5	5	2.3 ± 1.5	4	2.9 ± 1.5	4	<.001
Obesity	2.5 ± 1.7	5	3.4 ± 1.5	4	1.8 ± 1.5	5	2.8 ± 1.5	5	<.001
Physical exercise	2.5 ± 1.8	5	3.8 ± 1.4	3	1.6 ± 1.5	7	2.3 ± 1.6	8	<.001
Child bearing at a younger age	2.3 ± 1.7	7	2.8 ± 1.6	7	1.8 ± 1.5	5	2.5 ± 1.5	6	<.001
Alcohol	2.2 ± 1.8	8	3.1 ± 1.6	6	1.6 ± 1.5	7	2.5 ± 1.6	6	<.001
Risk factors for BC other than family history and HRT (sum)	15.0 ± 8.3		19.0 ± 8.1		12.0 ± 7.0		16.3 ± 7.4		<.001

BC: Breast Cancer/HRT: Hormonal Replacement Therapy.

*Adjusted for age, gender and specialty using multifactor ANOVAs.

### Preferred risk presentation formats (RPF)

In order to analyze this point, we opted for a five-cluster approach, which seemed to be the most relevant from both the statistical and clinical points of view (Table 4).

**Table 4:** **Clusters based on the risk information presented to patients – InCRisC (N=2094)**

	Formats used to communicate risk information							
	Numerical absolute risk n=1490 (71.2%)		Verbal formulation absolute risk n=893 (42.6%)		Numerical relative risk n=916 (43.7%)		Negative framing n=1398 (66.8%)	
	n	(%)	n	(%)	n	(%)	n	(%)
**Cluster 1: “No Verbal formulation & no Relative Risk mentioned” (n=368)**	368	(100.0)	0	(0.0)	0	(0.0)	368	(100.0)
**Cluster 2: “No negative framing” (n=717)**	479	(66.8)	249	(34.7)	280	(39.1)	21	(2.9)
**Cluster 3: “No Verbal Formulation” (n=267)**	267	(100.0)	0	(0.0)	267	(100.0)	267	(100.0)
**Cluster 4: “Risks presented in all 4 ways” (n=376)**	376	(100.0)	376	(100.0)	157	(41.8)	376	(100.0)
**Cluster 5: “Absolute numerical risks not presented” (n=366)**	0	(0.0)	268	(73.2)	212	(57.9)	366	(100.0)
GPs (n=1189)	830	(69.8)	475	(39.9)	463	(38.9)	743	(62.5)
BS (n=905)	660	(72.9)	418	(46.2)	453	(50.0)	655	(72.4)

The first cluster included 17.6% of the respondents. These respondents reported that they presented the risks in absolute numerical terms and in a negatively framed way, without any verbal description of the absolute risk and without mentioning the relative risks. This cluster was labelled “No verbal formulation & No relative risks mentioned”.

The second profile (34.2%) was that which occurred most frequently. It was characterized by the presentation of fairly heterogeneous information, and especially by the fact that only 2.9% stated that they would present negatively framed risks, i.e., the probability of not developing cancer. This cluster/profile was labelled “No negative framing”.

The third profile (12.7%) included practitioners who always presented the absolute numerical risks, the relative risks, and negatively framed risks. None of them described the absolute risks verbally. This group was labelled “No verbal formulation”.

In the fourth group (18.0%), the absolute risks were presented both numerically and verbally, and the risks were negatively framed. Nearly half of the members of this group also presented the relative risks. They were labelled as “Risks presented in all 4 ways”.

The last cluster (17.5%) included doctors who did not present the risks in absolute numerical terms and always framed them negatively. Most of this group presented the absolute risks verbally as well as presenting the relative risks. It was labelled “Absolute numerical risks not presented”.

### Characteristics of the clusters

The fourth cluster “Risks presented in all 4 ways”, which was taken to be one of the “best standards” for presenting risk information, was compared with each of the other clusters (Table 5).

**Table 5:** **Description of clusters in terms of physicians’ characteristics – InCRisC (N=2094)**

	Cluster 1, n=368 (17.6%)			Cluster 2, n=717 (34.2%)			Cluster 3, n=267 (12.7%)			Cluster 4, n=376 (18.0%)		Cluster 5, n=366 (17.5%)		
	Mean (SD)	AOR [CI 95%]	p	Mean (SD)	AOR [CI 95%]	p	Mean (SD)	AOR [CI 95%]	p	Mean (SD)	AOR	Mean (SD)	AOR [CI 95%]	p
Age	48.9 (9.1)	0.97 [0.96;0.99]	0.006	50.3 (8.9)	0.99 [0.98;1.01]	0.377	48.9 (8.4)	0.97 [0.96;0.99]	0.011	50.9 (8.7)	1	51.2 (8.5)	0.99 [0.98;1.01]	0.585
	n (%)			n (%)			n (%)			n (%)		n (%)		
Gender														
Female	156 (19.9)	1		258 (33.0)	1		88 (11.3)	1		133 (17.0)	1	147 (18.8)	1	
Male	212 (16.2)	0.91 [0.66;1.25]	0.564	459 (35.0)	0.98 [0.74;1.30]	0.886	179 (13.6)	1.18 [0.82;1.69]	0.374	243 (18.5)	1	219 (16.7)	0.81 [0.59;1.12]	0.207
Country														
France	62 (14.2)	1		186 (42.6)	1		71 (16.2)	1		55 (12.6)	1	63 (14.4)	1	
Germany	165 (18.2)	0.95 [0.62;1.46]	0.817	266 (29.3)	0.49 [0.34;0.70]	<.001	81 (8.9)	0.38 [0.24;0.59]	<.001	174 (19.2)	1	221 (23.4)	1.14 [0.75;1.73]	0.551
NL	48 (12.4)	0.53 [0.31;0.89]	0.016	144 (37.3)	0.54 [0.35;0.81]	0.003	63 (16.3)	0.65 [0.40;1.05]	0.081	74 (19.2)	1	57 (14.8)	0.63 [0.38;1.05]	0.076
UK	93 (25.5)	1.04 [0.64;1.70]	0.859	121 (33.2)	0.49 [0.32;0.75]	0.001	52 (14.3)	0.52 [0.31;0.86]	0.011	73 (20.1)	1	25 (6.9)	0.29 [0.16;0.51]	<.001
Speciality														
BS	144 (15.9)	1		258 (28.5)	1		147 (16.2)	1		195 (21.5)	1	161 (17.8)	1	
GPs	224 (18.8)	1.76 [1.31;2.38]	<.001	459 (38.6)	1.86 [1.44;2.41]	<.001	120 (10.1)	0.82 [0.59;1.13]	0.225	181 (15.2)	1	205 (17.2)	1.47 [1.09;1.98]	0.011

**Cluster 1: “No Verbal formulation & no Relative Risk mentioned” (n=368).**

**Cluster 2: “No negative framing” (n=717).**

**Cluster 3: “No Verbal Formulation” (n=267).**

**Cluster 4: “Risks presented in all 4 ways” (n=376).**

**Cluster 5: “Absolute numerical risks not presented” (n=366).**

In comparison with cluster 4, the respondents in cluster 1 “No verbal formulation & No relative risks mentioned” were more frequently GPs and belonged to a younger age-group, and were less frequently from the NTL.

In comparison with cluster 4, cluster 2 “No negative framing” contained a higher proportion of GPs and a higher proportion of French doctors.

In comparison with cluster 4, cluster 3 “No verbal formulation” included younger practitioners and a higher proportion of French doctors.

In comparison with cluster 4, cluster 5 “Absolute numerical risks not presented” more frequently included GPs and doctors practicing outside the UK.

To summarize, GPs were more frequently allotted to clusters corresponding to “incomplete” risk presentation formats; and the clusters brought to light some of the specificities of the countries under investigation (the NTL vs the others, France vs the others, UK vs the others). Although a younger age was associated with less verbal communication about the risks, gender was not found to be a significant determinant of the respondents’ risk communication profiles.

## Discussion and conclusion

This international survey is the first to assess how European GPs and specialised practitioners caring for healthy women under breast cancer surveillance regard their responsibility for assessing and communicating genetic, environmental and lifestyle breast cancer risk factors to their patients.

First, the majority of these European practitioners reported that they frequently assessed family history and hormonal replacement therapy (HRT) but only occasionally discussed patients’ lifestyle BC risk factors such as obesity, physical exercise and alcohol (Table 2). Breast surgeons communicated more frequently than GPs with their patients about all the breast cancer risk factors studied, except for HRT and oral contraception. Health providers from the Netherlands communicated much less frequently about patients’ lifestyle BC risk factors than those from the other countries, especially Germany, whereas French and UK doctors gave intermediate responses to this question.

The relatively similar attitudes to discussing patients’ family history and HRT risks observed in the various countries may have been due to the large body of research results published during the last fifteen years on the assessment of breast cancer genetic risks [7,12] and the deleterious effects of hormonal replacement therapy [23]. This knowledge has now been quite widely applied in the clinical practices of these practitioners, who seem to be convinced of their relevance as the result of either their initial/vocational training, the guidelines published or peer group recommendations. This was not found to be the case as far as lifestyle and changeable risk factors such as obesity, physical exercise and alcohol consumption are concerned. The importance of these factors has only been recognized quite recently, based on a lower level of evidence than with risk factors which have been studied in the framework of genetic testing and cancer genetic referrals, or HRT prescription in the context of menopausal symptoms. In addition, these factors are sometimes not even mentioned in the national guidelines for GPs and BSs. In view of the increasing evidence available that obesity, lack of physical exercise and probably high alcohol consumption rates contribute to breast cancer, there is certainly room for improvement in the guidelines published and physicians’ lifestyle recommendations to patients [24].

Secondly, the majority of the sample (more than two thirds) declared that they expressed the absolute risks numerically and framed them negatively when presenting them to patients (Table 4). Risks and relative risks were described verbally by only a minority of the respondents (less than 44%). The five profiles identified, corresponding to the preferred risk presentation formats (RPFs), differed in several respects: those of the relative majority of the respondents were incomplete, since they omitted to frame the cancer risks negatively, i.e., they did not mention the probability of not developing cancer (Cluster 2). Cluster 2 included more than one third of the sample and consisted more frequently of GPs and French practitioners, who expressed the opinion that they were responsible for explaining the risks as part of primary prevention and cancer screening efforts, but that they could not be expected to present a comprehensive picture of the risks. It has been recommended that healthcare providers should focus on the most relevant risks [14,15,19], and the respondents in cluster 2 obviously thought it was not relevant to talk about the chances of not developing cancer. This attitude might be judged to be inacceptable, since it is known to bias patients’ decision-making, which is contrary to the principle of patients’ freedom of choice and their right to express their own preferences. Cluster 4, “Risks presented in all 4 ways” along with cluster 3, “No verbal formulation”, which can be said to adhere most closely to the ‘best standards’ defined in the literature [14,15], differed in that cluster 3 more frequently consisted of younger doctors practicing in countries other than Germany/the UK. The “No verbal formulation & No relative risks mentioned” group could be said to consist of practitioners wanting to inform their patients quickly and simply about the risks, avoiding the difficult problem of explaining the relative risks, which has been found to help patients decide between the options available [19], but can be hard for patients to understand [15], as well as the rather delicate problem of presenting the risks verbally. On the other hand, risk presenters in the “Absolute numerical risks not presented” group could be said to show a more paternalistic attitude: making patients’ cancer risk perception as low as possible by presenting the absolute risk figures and emphasizing the probability of not developing the disease. These practitioners were more frequently GPs working in countries other than the UK. This paternalistic attitude is known to increase with age [25], and has often been observed mainly in male doctors [25]. This was not found to be the case here. The reason why the attitudes of the doctors surveyed here, as reflected in their risk presentation formats, were so variable may be that many of them had undergone very little formal training in the basic principles of risk communication [16] and that it is very difficult to apply theoretical recommendations in a real life context [19,26,27] in terms which can be easily understood by the patients [18,28].

This study has several limitations. First, it is about self-reported risk communication practices, which may differ from physicians’ actual practices. These declared practices are likely to be a combination between what the doctors said they would do and what they thought they ought to do. In addition, the vignettes used here provided only a short description of the complex clinical context. The results should be interpreted accordingly. Secondly, the questionnaire used in this study was mailed to the doctors eligible to participate, and the response rate obtained was low despite the reminders made by mail and telephone. The low response rate certainly reduce the possibility of generalizing the results obtained by taking them to be representative of doctors’ risk communication attitudes in a given country. Any country-specific differences will have to be investigated further. Although the response rate was low, it was comparable to that obtained in other surveys of this kind [29]. However, this survey is one of the few sources of information about an international sample of GPs’ and BSs’ self-reported cancer risk communication practices. It is of particular interest to note the different response rates obtained from one speciality and one country to another, since a low response rate can also be taken to reflect a low level of interest in the topic under investigation. It has been suggested that the most interested doctors are more likely to answer questionnaires and are therefore likely to be over-represented in the study sample.

The occupational characteristics of the practitioners working in various countries’ healthcare systems have to be taken into account when interpreting the data collected. The role of the “GP gatekeeper” in the UK’s and Netherlands’ healthcare systems differs from what occurs in France and Germany, especially as regards referrals, prescriptions and the existence of highly specialized secondary and tertiary care centres. For example, mammographic screening of healthy women at risk is performed in the UK by breast surgeons, whereas this activity is mostly carried out in France by Gynaeco-Obstetricians, who form a very heterogeneous group of practitioners, some of whom are medical gynaecologists (who do not carry out surgical interventions and deal very little with breast cancer patients), while others are involved only in obstetric care or specialize in breast surgery.

The differences between the breast cancer risk communication profiles observed among non-geneticist healthcare providers in these four European countries reflect the specificities of the respective healthcare systems, and especially the content of medical training programs and guidelines. Risk communication skills should be part of a core communication curriculum intended not only for geneticists/cancer geneticists and genetic counsellors, but also for primary care providers [30]. Qualitative studies might help to understand why practitioners adopt specific communication practices. The need for risk communication skills has been underestimated in healthcare providers’ initial and vocational training programs. In view of the current trend to investigate all the possible actionable scientific, medical and social risk factors involved in breast cancer, this topic would be worth investigating more closely.
